# Incidental extraspinal imaging findings on adult EOS full body radiographs: prevalence and clinical importance

**DOI:** 10.1186/s12880-021-00607-2

**Published:** 2021-05-17

**Authors:** Lily Wood, Christopher Martin, David Polly, Samuel Luchsinger, Takashi Takahashi

**Affiliations:** 1 Department of Orthopedic Surgery, University of Minnesota Medical School, Minneapolis, MN USA; 2 Department of Radiology, University of Minnesota Medical School, Minneapolis, MN USA

**Keywords:** Adult imaging, EOS imaging, Incidental findings

## Abstract

**Purpose:**

The purpose of this study was to review our institutional experience with the EOS machine in order to identify the incidence and clinical significance of incidental extraspinal findings (IESF) in an adult spinal deformity population.

**Methods:**

Our institutional database was queried for all full-length standing radiographs generated by the EOS machine. Dictations were reviewed and the number of incidental extraspinal findings were classified using a previously described system. All findings related to the spine were excluded. A subset of electronic medical records were reviewed to determine further workup for individual findings of suspected clinical significance.

**Results:**

Original database query based on radiology reports returned a total of 1857 EOS studies. Duplicate studies, studies without the entire body, and patients with more than 1 study during the search period were excluded. 503 patient studies (55.5% female, mean age 59-years-old, range 18 to 91-years-old) met inclusion criteria. The overall rate of incidental extraspinal findings in our study was 60.4% (304 findings in 503 patients). Most findings were classified as *Minor*. The rate of *Major* and *Moderate* findings was 4.8%. The final rate of clinically significant incidental extraspinal findings was 0.8% and included 3 presumed metastatic lesions in long bones and 1 pulmonary nodule.

**Conclusion:**

To our knowledge this is the first study that reports the rate of incidental extraspinal findings on full body EOS studies. We report a low rate (0.8%) of clinically significant incidental extraspinal findings which is lower than that of CT or MRI. Further research is warranted in comparing EOS and standard radiography.

## Background

**Fig. 1:**
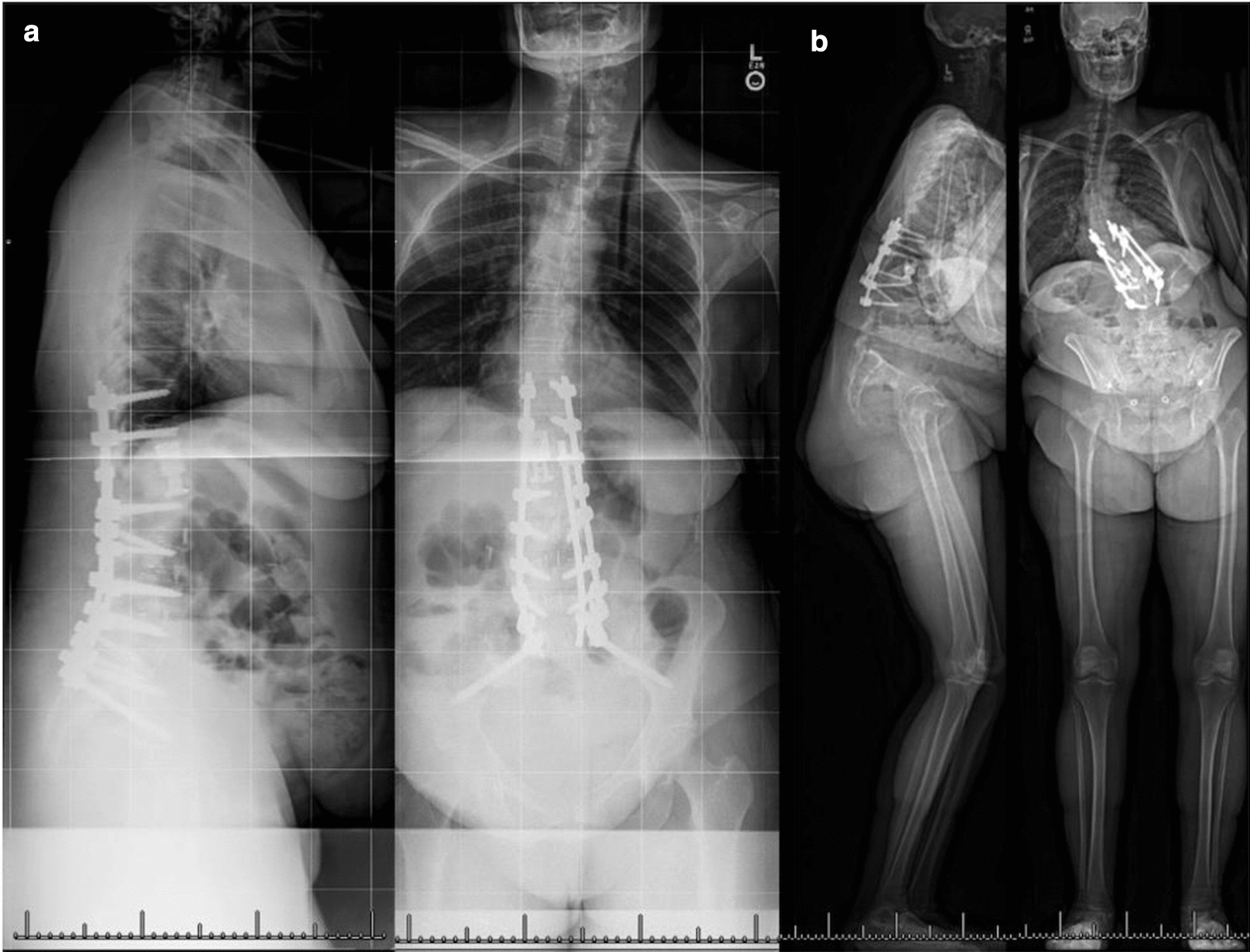
Traditional 36-inch plates (**a**) include only the axial skeleton, in contrast to EOS whole spine images, (**b**) which can include the entire skeleton

The evaluation of adult spinal deformity typically requires a full-length standing spine radiograph. Historically, these images were obtained using traditional plate radiography with 36-inch films (Fig. 1a). More recently, EOS technology (“EOS imaging”, Paris, France) has allowed simultaneous capture of biplanar imaging in an upright position, to include the entire skeleton (Fig. 1b). This technology carries some advantages for the spinal deformity surgeon including accurate assessment of global spine and skeletal standing alignment, including an assessment of leg length and position. Additional benefits include reduced radiation exposure [1–6] and the generation of 3D reconstructions from 2D images using the sterEOS platform that may be of comparable quality to CT reconstructions [3]. However, disadvantages include investment and operating costs [2], including for sterEOS software, as well as potentially inferior resolution quality, as compared to standard radiography [7, 8].

The inclusion of the entire skeleton and soft tissue envelope, potentially allows for the detection of incidental extra-spinal findings (IESF). Incidental findings by definition are any abnormality, detected in the context of medical diagnostics, that may affect the health of the individual and are not related to the original indication for conducting the study [9, 10]. As more imaging studies are performed, at higher resolution, the number of reported incidental findings has increased [11]. The incidence and impact of incidental findings in other imaging modalities, particularly for magnetic resonance imaging (MRI) [12, 13], and computed tomography (CT) studies [14, 15], has been well established, with up to 68% of high resolution axial images showing at least one incidental finding [12]. However, given the relatively recent introduction of the EOS technology, its impact on the detection of incidental findings has not been well established, and to our knowledge no prior studies have reported the incidence of incidentalomas on this imaging modality.

The detection and reporting of incidental imaging findings has significant implications both for radiologists and surgeons [16]. At our own institution the surgeons previously read their own spinal deformity radiographs, without requesting a radiologist interpretation. However, in part due to fear of missing an IESF, radiologists now comment on every deformity study. Without knowing how often incidentalomas are found, it is difficult to know how helpful this policy is. Thus, the purpose of this study was to review our institutional experience with the EOS machine in order to identify the incidence and clinical significance of IESF in an adult spinal deformity population.

## Methods

### Subjects

Our institution maintains a searchable database of radiologists’ dictations. All full-length standing radiographs taken on the EOS machine are dictated with a standard template, which includes the term, “AP and lateral EOS composite images of full body.” The imaging and dictation template came into standard use in the fall of 2017, and thus we chose that time frame for our study period. After obtaining institutional IRB exemption, we retrospectively queried the database in order to identify all full-length, standing radiographs taken between October 2017 and September 2018. Only adult patients (> 18-years-old) were included. The initial list was then reviewed by a study team member (LW) who read the radiologists’ dictations and applied our inclusion criteria. We excluded additional images linked to the imaging study of interest (e.g. individual studies of the “lumbar spine only”), as well as “full spine only” studies, and “lower extremity only” studies. Some patients were imaged multiple times during the study period. If a patient was imaged multiple times we used only the first study performed. This exclusion criteria prevents over-counting the number of IESF.

### Classification

For each included study, one of our study team members (LW) reviewed the radiologist dictation. All findings beyond the axial spine—incidental extra-spinal findings (IESF)—were then recorded and classified by body system (e.g. pulmonary, vascular, bony systems). Findings related specifically to the spine and/or spinal surgery were intentionally not included. Although our ability to determine if a finding occurred secondary to a surgical procedure is dependent upon accurate and thorough dictations and documentation in the electronic medical record.

**Fig. 2:**
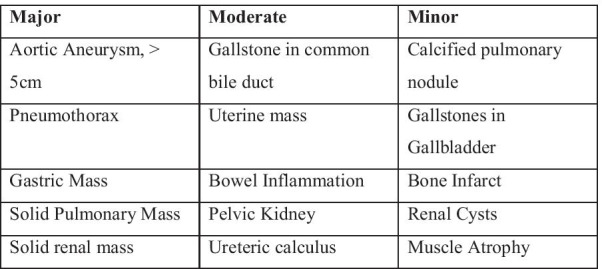
Classification of IESF on body imaging, adapted from The Royal College of Radiologists [21]. Major—always requires further workup and likely to have adverse health effects. Moderate—may require further workup but health significance is unclear. Minor—unlikely to require further workup or have significant health effect

A variety of methods exist for classifying incidental findings [10, 11, 17–21]. For the purposes of our study we chose to classify the IESF according to the framework developed by The Royal College of Radiologists (RCR) (Fig. 2). The use of three tiers of findings—Major, Moderate, and Minor—subdivides IESFs based on their potential clinical implications [20]. *Major findings* include abdominal aortic aneurysms and findings concerning for malignancy and always require further investigation and are likely to have adverse health effects. *Moderate findings* include splenomegaly and usually require further intervention but have unclear health effects. *Minor findings* are innately nonsignificant and lack clear prognostic and/or clinical consequence. Because pulmonary nodules do not fit nicely into one of the RCR’s categories we chose to augment the classification using the 2017 Fleischner Criteria [22] and classified all non-calcified nodules > 8 mm as *Major* and all nodules < 8 mm, *Moderate*. Any calcified nodule was expected to be a granuloma and considered *Minor* in keeping with the RCR criteria.

**Fig. 3:**
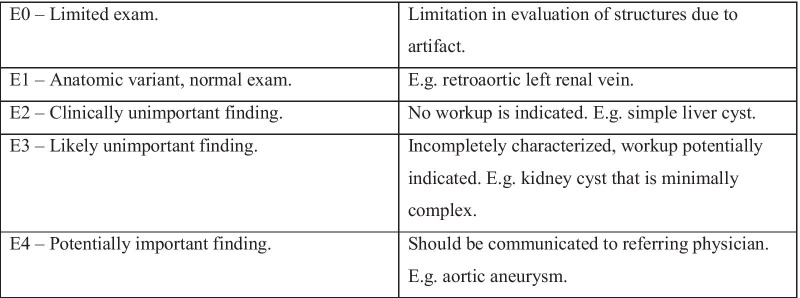
CT colongraphy reporting and data system (C-RADS) categorization and management recommendations for extracolonic findings

In order to compare our work to that of other authors [12, 13, 23] we also secondarily classified the IESF using the CT Colonography Reporting and Data System (C-RADS) [21] (Fig. 3). C-RADS was developed to standardize a reporting of extra-colonic incidental findings as well as also offer recommendations on management of lesions [21].

### Clinical significance of IESF

Once initial classification had been made, the electronic medical record (EMR) was reviewed for all *Moderate* and *Major* IESF in order to determine the subsequent clinical follow-up and thus clinical significance of these findings. We define clinically significant IESF as any finding that required further intervention (e.g. ongoing follow up with a specialist, additional imaging on a regular basis, etc.). IESF that were determined on subsequent workup to be *Minor* findings (i.e. a nodule that is revealed to be a granuloma on CT) were classified as “Insignificant.” This category contains false positives or what may be thought of as “overcalls.” Findings that were not further assessed were categorized as “no follow up.” This category likely include those IESF deemed, without documentation, clinically insignificant by the clinician ordering the study as well as recommendations lost in the course of communication.

## Results

### Patient population

**Fig. 4:**
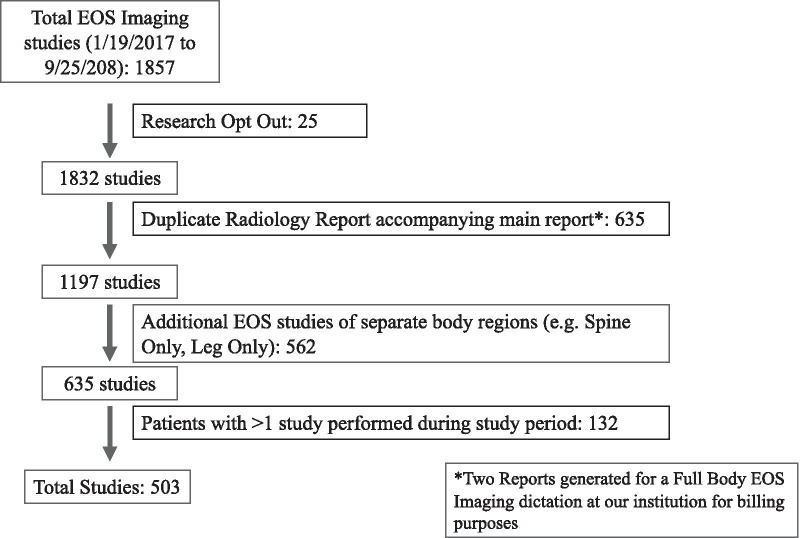
Inclusion and exclusion criteria and patient selection

The initial institutional radiology report database query from 1/19/2017 to 9/25/2018 identified 1857 radiology reports. Once exclusion criteria were applied a total of 503 unique patient studies were included in our analysis (Fig. 4). Of the 503 patients, 279 were female (55.5%) and 224 were male (44.5%), with an average age of 59 (58.5) years (Range 18–91 years-old, SD 15.8) (Table 1).

**Table 1:** Demographics

Characteristic	Value
Average age (years)	58.5
Range (years)	18–91
Standard Deviation (years)	15.8
Sex, (male/female)	224 (44.5%)/279 (55.5%)
Total (n)	503

*SD* standard deviation

### Incidental findings

In total there were 260 extra-spinal degenerative findings, 9 patients with femoro-acetabular impingement (FAI), 3 patients with extra-pulmonary thoracic findings, 14 patients with pulmonary findings, 3 patients with vascular findings, 2 patients with abdominal findings, 12 patients with bony abnormalities, and 1 patient with a finding we categorized as “Other”, for a total of 304 IESF. In total there were 280 *Minor* findings, 18 *Moderate*, and 6 *Major* findings (Table 2). Secondary comparison of the IESF to the C-RADS [21] classification system is presented in Table 3. A summary of each of the IESF by system is reported below.

**Table 2:** Categorization of IESF by RCR classification

	Major (n)	Moderate (n)	Minor (n)
Degenerative change (260)			260
Femoroacetabular impingement (FAI) (9)			9
Thoracic, Extra-Pulmonary (3)			
Paratracheal fullness		1	
Soft tissue density		1	
Widened mediastinum		1	
Pulmonary (14)			
Pulmonary nodules, solid, not calcified (> 8 mm)	3		
Pulmonary nodules, solid, not calcified (< 8 mm)		2	
Granuloma			6
Pleural effusion			1
Pleural plaque			1
Nodule v. Artifact		1	
Abdominal (2)			
Calcification		2	
Vascular (3)			
Enlarged/prominent cardiac silhouette		3	
Bony abnormalities (12)			
Bone infarct	3		
Lytic bone lesion			3
Sclerotic bone lesion		2	
Rib abnormality		3	
Hardware loosening		1	
Other (1)			
VP Shunt Discontinuity v. artifact		1

**Table 3:** Comparison of RCR v. C-RADS classification of IESF

RCR/C-RADS	E2 (n)	E3 (n)	E4 (n)
Minor	280		
Moderate		18	
Major		3	3

Note that the classification systems are mostly concordant besides three pulmonary nodules that were classified as *Major* per RCR criteria and E3 per C-RADS criteria (based on size)

#### Degenerative change

260 patients (149 female, 53.8%, average age 64-years-old) had reported degenerative changes in extra-spinal joints including knees, hips, carpometacarpal (CMC) joints, ankles, glenohumeral joints, and acromioclavicular joints. All degenerative change was categorized as *Minor* (see Table 2) and only one area of degenerative change was counted per patient even if more than one joint had evidence of degenerative change on the EOS study. Radiologists did not recommend follow up for any of the findings of degenerative change.

#### Femoroacetabular impingement (FAI)

9 patients (3 female, 30%, average age 31.5-years-old) had a dictated finding of femoro-acetabular impingement, such as abnormal femoral neck morphology, femoral neck-head offset, or CAM type lesions of the femoral neck. All FAI findings were categorized as *Minor*. Any patient over 50-years-old with dictation of FAI was not included. Radiologists did not recommend follow up for any of the FAI findings.

#### Thoracic, extra-pulmonary abnormalities

3 patients (1 female, 33%, average age 66-years-old) had a dictated finding of thoracic, extra-pulmonary abnormalities including paratracheal fullness, widened mediastinum, and supra-hilar density. All findings were classified as *Moderate*. All dictations recommended follow up imaging.

#### Pulmonary abnormalities

14 patients (8 female, 57%, average age 64-years-old) had a dictated finding of pulmonary abnormality including granuloma (6), pulmonary nodule (4), multiple nodules v. apical thickening (1), pleural plaque (1), pleural effusion (1), and pulmonary nodule v. artifact (1). All granulomas, pleural effusion, and pleural plaque were classified as *Minor*. 3 pulmonary nodules were 9, 9, and 10 mm, respectively, and, classified as *Major*, and the other one nodule was 5 mm and classified as *Moderate*. The abnormality “apical thickening v. pulmonary nodule” was 4–5 mm and also classified as *Moderate*. Finally, the nodule v. artifact was classified as *Moderate.* All dictations for *Moderate* and *Major* findings recommended follow up imaging.

#### Vascular abnormalities

3 patients (3 female, 100%, average age 65-years-old) had a dictated finding of vascular abnormality. All 3 vascular abnormalities were described as enlarged or prominent cardiac silhouettes. All 3 were listed as *Moderate.* 2 of the 3 dictations recommend follow up imaging. Of note, vascular calcifications were not included in our study.

#### Abdominal abnormalities

2 patients (1 female, 50%, average age 62-years-old) had a finding of non-vascular calcification located within the abdomen. Both were classified as *Moderate* and both dictations recommended follow up.

#### Bony abnormalities

12 patients (7 female, 58.3%, average age 61-years-old) had a dictated finding of bony abnormality including bone infarct (3), lytic/lucent long bone lesion (3), sclerotic long bone lesion (2), rib abnormality (3) or loosening of hardware (1). All lytic lesions were classified as *Major*, all bone infarcts categorized as *Minor*, and all other findings classified as *Moderate*. Dictations recommended follow up in 2 of 3 *Minor* findings, all *Moderate* and 2 of 3 *Major* findings.

#### Other abnormality

1 patient (1 female, 100%, age 50-years-old) had a dictated finding of possible discontinuity in a ventriculoperitoneal (VP) shunt. The dictation recommended clinical correlation. The finding was classified as *Moderate* because of significant implications if the shunt was indeed in discontinuity.

### Clinical significance

We reviewed the electronic medical record (EMR) of patients with 24 IESF (i.e. classified as *Moderate* or *Major*) (Table 3)*.* We did not review the EMR for any *Minor* IESF because, by definition, these findings are not clinically significant and unlikely to have adverse health effects for the patient. Therefore, we did not review the EMR for the following abnormalities: degenerative change, FAI, pleural plaque, pleural effusion, granuloma, or bone infarct. The clinical significance of these findings in our study is unknown.

One of the 3 thoracic, extra-pulmonary findings was followed up with additional imaging. The paratracheal fullness, concerning for mediastinal lymphadenopathy, on further chest X-ray and CT was determined to be without corresponding finding and classified as “Insignificant”.

**Fig. 5:**
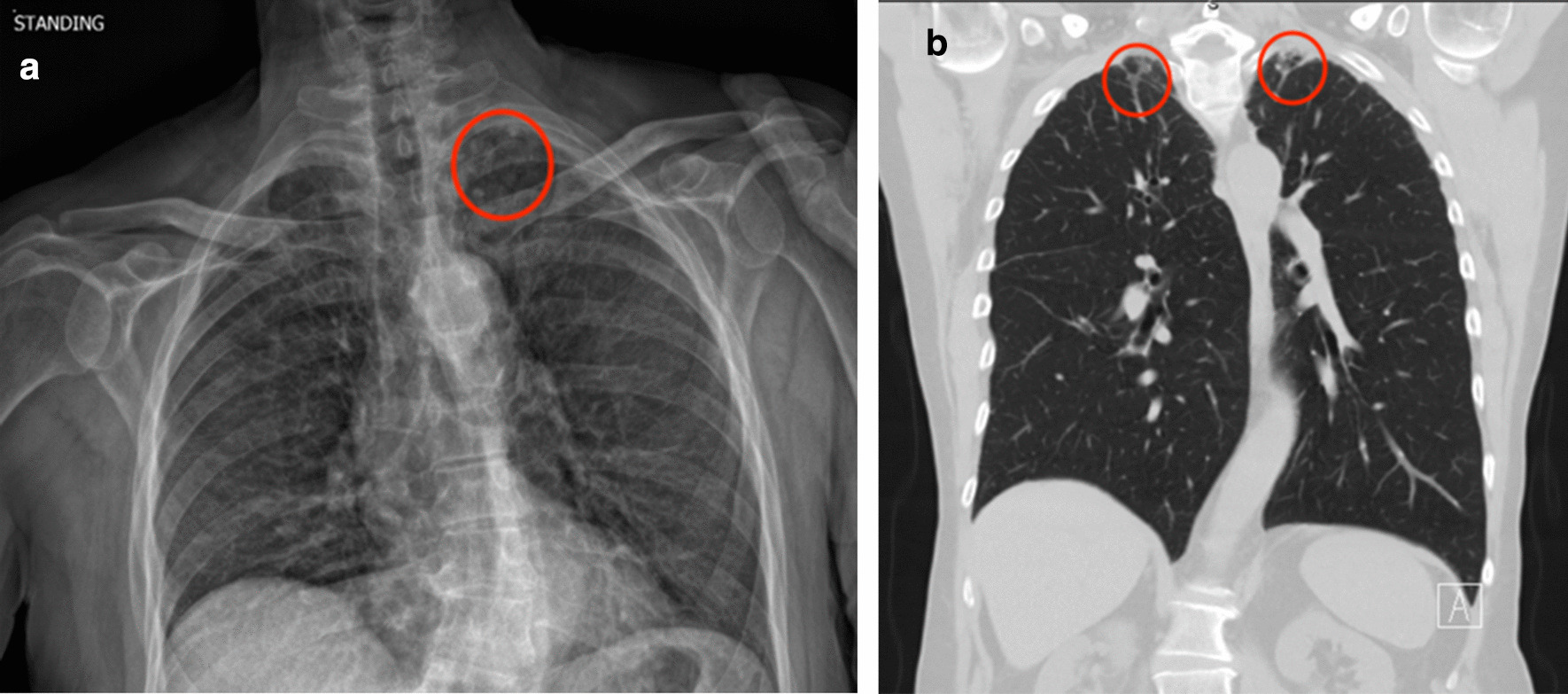
**a** A 70-year-old man with 4–5 mm left apical pulmonary nodules (circle) (a *Moderate* finding) on EOS imaging. **b** The same patient with subsequent coronal reconstruction of CT chest using a lung window showing bi-apical subpleural reticulonodular opacities (circles), raising concern for possible interstitial lung disease (ILD)

All 3 *Major* pulmonary nodules were classified as Insignificant after further workup. 2 were referred for CT studies and determined to not require additional intervention. The other *Major* nodule was known to the patient and the imaging was sent to their primary care provider at an outside facility. Of the 3 *Moderate* findings, 1 was determined to be a granuloma (Insignificant) by an outside provider using an older CT study. The finding of nodule v. artifact was never followed up, per chart review. The patient with 4–5 mm nodules (Fig. 5a) was referred to a pulmonologist who diagnosed possible Interstitial Lung Disease (ILD) and continued to see the patient for 2 years of regular follow up and imaging (Fig. 5b shows follow up CT chest of nodules). This finding was deemed “Significant”.

Of the Vascular IESF, 3 enlarged cardiac silhouettes were classified as *Moderate*, and none of the 3 were further investigated (No follow up), per the EMR.

Of the Abdominal IESF, 1 finding was not further followed up. The other finding was thought to be a renal abnormality and additional studies including MR renal and ultrasonography resulted in diagnosis of a simple, exophytic renal cyst (Insignificant).

**Fig. 6:**
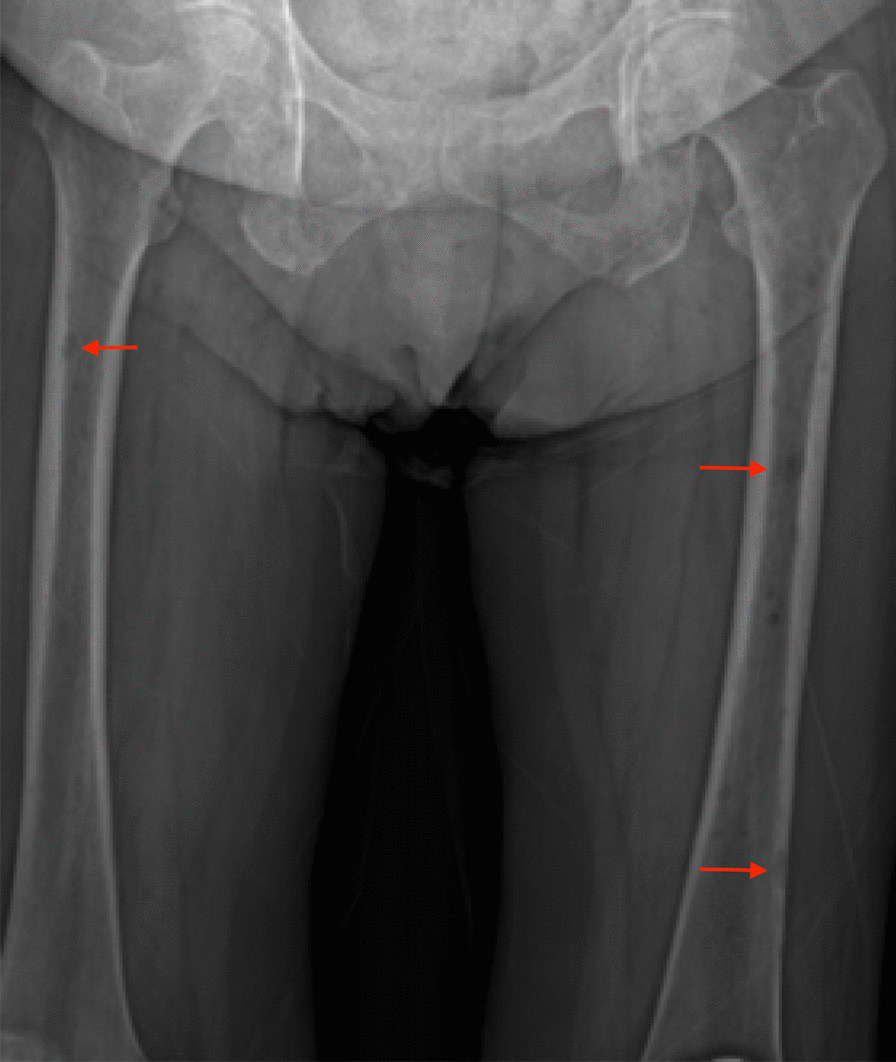
A 71-year-old woman with history of metastatic breast cancer with multiple lytic lesions (arrows) (a *Major* finding) on EOS imaging

Of the Bony IESF, all 3 lytic bone lesions occurred in patients with history of metastatic cancer (Significant) although the findings were not followed up with further imaging, presumably because the patients’ clinicians were already aware of the presence of metastatic disease. Figure 6 is a magnified EOS image of one of the 3 patients with lytic lucencies. One of the 2 sclerotic lesions was not followed up while the other was evaluated by orthopedic providers (including an orthopedic oncologist) and determined to be a Non-ossifying fibroma (Insignificant). As for the 3 rib abnormalities, 1 received no further workup while 2 did. One rib lesion was followed up with CT chest at outside facility and determined to be consistent with old trauma (Insignificant) and the other was followed up with CT chest at our facility and no specific clinical action was taken (Insignificant). The finding of hardware loosening was in the femoral component of a total hip replacement and was not followed up.

The term “Other” was used to classify a dictation with concern for discontinuity of a VP shunt. The patient presented to neurosurgery clinic, for a different reason, after their EOS study where the shunt was evaluated by providers without mention of abnormality (Insignificant).

Comparison of IESF between the RCR and C-RADS classification systems yields mostly concordant categorizations. Although, 3 pulmonary nodules classified as *Major* according to our modified Fleischner and RCR criteria were classified as “E3” findings because the nodules were less than 1 cm.

### Overall

The overall rate of IESF for our study (including all *Minor*, *Moderate* and *Major* findings) was 60% (60.4). The rate of clinically significant IESF was 0.8% and consisted of 1 pulmonary nodule and 3 lytic bone lesions. 24 IESF (4.8%) were classified as either *Major* or *Moderate* and were recommended by radiologist dictations for further workup. 14 of these 24 IESF (58.3%) were further investigated, per review of the EMR, with either additional imaging, discussion amongst providers, or referral to a subspecialist. There was no evidence in the medical record that the remaining 10 findings were further evaluated at our institution or at an outside institution. The rate of false positives (i.e. Insignificant column in Table 4) was 42%.

**Table 4:** Clinical significance of major and moderate IESF

	Significant (n)	Insignificant (n)	No Follow Up (n)
Thoracic, Extra-Pulmonary (3)			
Paratracheal fullness		1	
Soft tissue density			1
Widened mediastinum			1
Pulmonary (6)			
Pulmonary nodules, solid, not calcified (> 8 mm)	1	3	
Pulmonary nodules, solid, not calcified (< 8 mm)	1	1	
Nodule v. Artifact			1
Abdominal (2)			
Calcification			1
Renal cyst		1	
Vascular (3)			
Enlarged/prominent cardiac silhouette			3
Bony Abnormalities (9)			
Lytic bone lesion	3		
Sclerotic bone lesion		1	1
Rib lesion		2	1
Hardware loosening			1
Other (1)			
VP Shunt Discontinuity v. artifact		1	

## Discussion

Incidental imaging findings can occasionally result in life saving information, which has significant ethical implications for the patient [16]. In part, this concern has encouraged our institution to request a radiologist comment on every radiographic study. Numerous reports have identified the incidence and significance of incidentalomas on CT [23, 24] or MRI-based [12, 13] spine studies, but the incidence for EOS full body imaging has not been well established. Here, we identified a high rate of benign radiographic findings and a low rate of clinically significant findings on whole body EOS images taken in a spinal deformity population.

One of the main challenges of this study was comparing our data to that of other authors due to the variety of systems used to classify incidental findings (Table 5). Given the challenges of categorization, radiology organizations have responded by publishing recommendations on the classification and management of incidental findings [9, 20]. To this end, guidelines such as the Fleischner Criteria for classification and management of pulmonary nodules identified on CT [22], and the White Papers, from the American College of Radiology [25], have been published. Although, even when express guidelines exist, some authors find that radiologists may continue to make their own, individualized recommendations regarding further workup for a given finding [17]. More inclusive, intentionally nonspecific, classification systems have been developed by other authors while performing retrospective studies and reviews of the literature on incidental findings [17, 18, 20]. However, we still found discrepancies in terminology and consistency in application.

**Table 5:** Comparisons of IESF for studies

Study	Study characteristics	Total Pts (n)	Age	“Nodule” rate	Clinically relevant IESF	Total IF rate
Our study	EOS imaging IESF rate	503 44.5% male	Mean 60 yo Range: 18–91 yo	Nodules, only: 0.99% Nodules + granulomas 2.2%	Major and Moderate: 4.8% Clinically relevant: 0.8%	60.4%
Den Harder et al. [31]	Cardiac patients undergoing preop CXR IF rate Included spinal abnormalities	1136 70% male	Mean: 65 yo No Range reported	Pulm mass: 0.8%	Findings that resulted in further workup: 1.3%	50% (patients that had 1 + abnormalities on CXR)
Van Vugt et al. [32]	European study, multicenter CXR for acute cough in outpatient clinic IF rate	2823 43.5% male	Mean: 53 yo Range: 18–92 yo	Nodule, density shadow rate: 1.8%	Clinically relevant: 3%	Mean: 19% Range (from individual centers): 0–25%
Quattrochi et al. [12]	Lumbar MRI (1.5 T) IESF rate No thoracic findings in study	3000 48.4% male	Mean: 59.3 yo Range: 16–91 yo	–	E3 + E4: 11.3% 16.5% of patients E4: 2.5% E2: 57.4%	68.6%
Semaan et al. [13]	Lumbar MRI IESF rate	3024 45% male	Mean: 63 yo Range: 18–95 yo	–	E3 + E4: 6.65% E2: 20% E4: 1.25%	22%
Lee et al. [24]	CT lumbar spine for LBP IESF rate	400 53% male	Mean: 49 yo Range: 20–91 yo	–	E3 + E4: 14.8% E4: 4.3% E2: 25.3%	40.5%

*IF* incidental finding, *IESF* incidental extraspinal finding, *CXR* chest radiograph, *MRI* magnetic resonance imaging, *CT* Computed Tomography

One system that has been utilized previously when classifying IESF on CT and MRI studies is the CT Colonography Reporting and Data System (C-RADS) [21]. Adapting this nomenclature, E2 approximates our term *Minor*, E3 approximates *Moderate*, and E4, *Major*. However, one distinct difference is that pulmonary masses > 1 cm represent an E4 finding whereas we used a cutoff of > 8 mm (as in our “Methods” section). Overall, as shown in Table 3, our classification does mostly agree with the C-RADS system (besides 3 pulmonary nodules that were smaller than the cutoff of 1 cm) which means both systems are comparable for comparison and classification purposes; this was one welcome consistency since, as discussed above, classification of incidental findings is generally challenging. Prior studies of lumbar spine CT report a rate of total E3 and E4 findings (indeterminate and clinically important findings) of 14.8% [23] whereas we calculated a rate of 4.8%. This rate is higher than what we calculated for our *Moderate* and *Major* findings (4.8%) even though the field of view for the above studies was limited to the lumbar spine. However, this may be expected given the higher contrast resolution of CT over radiography. For example, these authors report high rates of vascular aneurysms and organomegaly which would have been much harder to detect on radiography.

Similarly, the rates of E3 and E4 IESF on lumbar spine MRI studies is reported to be between 7.8% [13] and 12.0% [12] which is higher than our rate (again, 4.8%) but on par with that of CT lumbar spine studies [23]. These large MRI studies of 3000 + patients report high rates of fluid collections, enlarged lymph nodes, and aortic aneurysms which, as previously stated, would have been difficult to capture with EOS imaging.

Rather than comparing EOS to CT and MRI, one might draw better comparisons between EOS and other standardized radiography modalities, including X-ray (i.e. computed radiography, digital radiography and traditional X-ray). Authors generally agree that one of the main benefits of EOS is decreased radiation exposure at skin surfaces [1, 3–5] on whole spine and body imaging, especially when micro-dose protocols are instituted [5], as compared to standardized radiography. What appears to be less generally agreed upon is the quality of the 2D EOS images. Previously published studies report accurate reconstruction of bony structures [26, 27] as well as highly reproducible measurements, including limb length measurements [28] and 3D reconstructions of bony structures [29] including pre and postoperative imaging [30] and classification and identification of lumbosacral vertebrae [31]. Some authors suggest that resolution and quality of images is inferior to traditional X-ray modalities [2, 7]. Alternatively, a systematic review reported on three studies comparing EOS versus X-ray imaging (film or Fuji FCR 7501S) and found that the image quality of EOS was comparable if not better than the other standard X-ray modalities [6]. The authors did note that there were differences in the study methodologies and reporting of data, and, overall, there was a paucity of existing current literature comparing EOS to standard X-ray.

EOS imaging is an asset to the spinal deformity surgeon who is globally evaluating spinal deformity. However, based on limitations in imaging quality, EOS may be inferior to traditional forms of radiography in evaluation of incidental findings. Therefore, we attempted to compare our rates of IESF on EOS imaging to rates of IESF on standardized X-ray. At one university hospital, chest radiographs are a part of the preoperative protocol for patients undergoing cardiac surgery. Here, the authors report 50% of patients had at least one incidental finding, although these findings included vascular calcifications and spine abnormalities, both of which we excluded in our analysis [32]. 1.3% of patients underwent further workup of incidental findings which revealed one pulmonary mass for a rate of 0.08%. Unfortunately, the authors did not define criteria for a pulmonary mass. Our rate of pulmonary nodules (> 6 mm, non-granuloma) was higher (0.99%), which may be attributable to many factors, including differences in patient demographics (cardiac patient cohort, mean age 65-years-old, and mostly male), differences in classification of pulmonary findings, and/or differences in imaging modality. Another multi-center study in Europe assessed the rate of incidental findings in patients (mean age 50-years-old, 40% male) undergoing chest radiographs for acute cough. They report a rate of “clinically relevant” findings (comparable to our *Major* and *Moderate* findings) of 3.1% (we report 4.8%) and report a rate of nodules, densities, and shadows of 1.8% [33]. Our nodule rate (including granulomas) was 2.2% but excluding granulomas is 0.99%. The authors also found higher rates of cardiomegaly (3.6%), mediastinal enlargement (1.0%), and pleural abnormalities (1.4%) which suggests that chest radiographs, perhaps due to the smaller field of view and potentially better resolution, may pick up thoracic abnormalities better than EOS. Overall, there is not enough evidence in the literature and certainly not enough evidence here, to confidently state that either EOS or standard radiography are better at detecting incidental findings.

This study has several limitations. One limitation is that if patients had multiple EOS studies performed during the study period we used the first EOS imaging study (by date) only. We assumed that patients were unlikely to have significant changes in incidental findings over time. However, there is a possibility that we may have missed incidental findings due to a change in the patient’s condition—i.e. weight change, surgical changes, etc.—that would have augmented the radiologists’ ability to detect an IESF on follow up imaging resulting in either over or underestimating the total IESF in our study. Furthermore, essentially all whole-body EOS studies at our institution were ordered by spine surgeons; hence, there is a selection bias of the study population with a higher incidence of either corrected or uncorrected underlying spinal deformity. For example, thoracic hyperkyphosis may decrease identification accuracy of chest findings. However, we believe this is an acceptable limitation as whole-body EOS studies are indeed most commonly used for this specific target population. Additionally, the current study includes all incidental findings reported by radiologists and may include findings that were previously known to providers outside of the patient’s care team at our institution. We did review the charts of all the significant incidental findings in our study, as previously described, but there is a chance that, for example, a finding of FAI may have been previously noted or even evaluated by a provider which was not apparent using our methods of review. Another limitation of our study is that we only reviewed the original radiologist dictation, and did not re-review the imaging studies themselves. It is possible that re-review of the EOS images would have identified a higher incidence of incidental findings, and indeed some prior studies have found this to be true for MRI based studies [13]. Investigating the “nondetection rate” is a potential future direction for our department.

### Conclusion

In conclusion, what the ordering surgeon really wants to know is, “Are there any extra-spinal findings with clinical significance that require follow-up?” And the radiologist wants to know, “Are there extra-spinal findings of radiographic significance that may warrant further intervention by clinicians?” To both questions we would say, “yes.” We report a low rate of clinically significant pulmonary and bony findings (including 3 presumed metastatic lesions and 1 pulmonary nodule likely to be ILD) on EOS imaging. The rate is low which may be attributable to inferior imaging capabilities of EOS as compared to standard x-ray studies.

## Data Availability

The datasets used and/or analyzed during the current study is available from the corresponding author on reasonable request.
